# Fossilized anuran soft tissues reveal a new taphonomic model for the Eocene Geiseltal Konservat-Lagerstätte, Germany

**DOI:** 10.1038/s41598-024-55822-y

**Published:** 2024-04-23

**Authors:** Daniel Falk, Oliver Wings, Richard Unitt, Jon Wade, Maria E. McNamara

**Affiliations:** 1 School of Biological, Earth and Environmental Sciences, University College Cork, Distillery Fields, North Mall, Cork, T23 TK30 Ireland; 2 Environmental Research Institute, University College Cork, Lee Rd, Cork, T23 XE10 Ireland; 3 Natural History Museum Bamberg, Staatliche Naturwissenschaftliche Sammlungen Bayerns, Fleischstraße 2, 96047 Bamberg, Germany; 4 Copper Coast UNESCO Global Geopark, Knockmahon, Bunmahon, X42 T923 Ireland; 5 Department of Earth Sciences, University of Oxford, South Parks Road, Oxford, OX1 3AN UK

**Keywords:** Palaeoecology, Palaeontology, Sedimentology, Geochemistry, Biogeochemistry

## Abstract

The Eocene Geiseltal Konservat-Lagerstätte (Germany) is famous for reports of three dimensionally preserved soft tissues with sub-cellular detail. The proposed mode of preservation, direct replication in silica, is not known in other fossils and has not been verified using modern approaches. Here, we investigated the taphonomy of the Geiseltal anurans using diverse microbeam imaging and chemical analytical techniques. Our analyses confirm the preservation of soft tissues in all body regions but fail to yield evidence for silicified soft tissues. Instead, the anuran soft tissues are preserved as two layers that differ in microstructure and composition. Layer 1 comprises sulfur-rich carbonaceous microbodies interpreted as melanosomes. Layer 2 comprises the mid-dermal Eberth–Katschenko layer, preserved in calcium phosphate. In addition, patches of original aragonite crystals define the former position of the endolymphatic sac. The primary modes of soft tissue preservation are therefore sulfurization of melanosomes and phosphatization of more labile soft tissues, i.e., skin. This is consistent with the taphonomy of vertebrates in many other Konservat-Lagerstätten. These findings emphasize an emerging model for pervasive preservation of vertebrate soft tissues via melanosome films, particularly in stagnation-type deposits, with phosphatization of more labile tissues where tissue biochemistry is favorable.

## Introduction

Konservat-Lagerstätten are characterized by high fidelity preservation of fossils as articulated skeletons and/or with soft tissues^1,2^. Such fossils provide critical insights into the evolution of extant plants and animals^2,3^. Konservat-Lagerstätten from the Paleogene and Neogene are of particular interest as they typically have experienced less complex diagenetic histories^4^, including less thermal maturation, than their geologically older counterparts. As a result, the pathways leading to preservation are usually not completely obscured by chemical alteration and/or deformation. Further, fossils from Paleogene and Neogene Konservat-Lagerstätten are usually anatomically similar to extant analogues^4^. Models for preservation developed using such fossils can therefore be applied to fossils from older Konservat-Lagerstätten in order to inform interpretations of preserved anatomy, chemistry, and the controls on preservational fidelity.

Notable Paleogene Konservat-Lagerstätten include the Geiseltal biota of central Germany (late early to middle Eocene^5,6^), which is the subject of renewed interest due to the recent reopening of the collections for scientific access^7^. The biota is represented by ca. 50,000 specimens, more than half of which are vertebrates, including amphibians, fish, lizards, mammals, and birds^7,8^. Many vertebrates preserve soft tissues, evident in hand specimens as pale- to brown-toned body outlines^8–11^. The preserved soft tissues were first investigated in the 1930s^12–16^ (reviewed in Voigt^10^). These studies reported the preservation of muscle tissue (in artiodactyls, bats, fish, frogs, lizards and palaeohippids), cartilage (in bats, newts, palaeohippids and reptiles), blood vessels with erythrocytes (in frogs and lizards), integument (bat- and frog skin, lizard scales, feathers and hair of artiodactyls, marsupials, and palaeohippids), connective tissue (in bats, crocodiles, fish, frogs, lizards) and calcium carbonate crystals of the endolymphatic sac (in frogs). Pigment cells were reported in bats (skin), fish (eyes and skin), newts (skin) and frogs (skin)^10,16^; “Melaninkörnchen” [melanin granules] (Voigt^16^, p. 9; Voigt^10^, p. 328) and/or “Pigmentkörnchen” [pigment granules] (Voigt^16^, p. 5; Voigt^10^, p. 336) were reported in fish (eyes and skin), frogs (skin) and marsupials (hair; Voigt^10^, p. 328). Fossil bacteria were reported in fish, frogs and the coprolites of crocodiles^10,17^. In addition, certain specimens of artiodactyls and perissodactyls preserve stomach contents^10,11,14^. Collectively, these reports of subcellular anatomical details for various soft tissues in different vertebrate groups describe a fidelity of preservation that is among the highest known for any Lagerstätte. Ultrastructural preservation of soft tissues with comparable fidelity is known for other fossil vertebrates from Neogene^18,19^ and Cretaceous biotas^20^, but is often restricted to a limited number of taxa.

The proposed mode of preservation of the Geiseltal fossils, i.e., direct three-dimensional replication in silica^10,16^, is not known elsewhere in the fossil record. The evidence for this mode of preservation therefore warrants consideration and validation. The fidelity of anatomical preservation of soft tissues in Geiseltal fossils was originally assessed using transmitted light microscopy of whole tissue samples (i.e., not tissue sections)^10,12–14,16^. These studies also investigated the chemistry of the soft tissues by combustion of tissue samples and analysis of the residues using refractometry^16^. Based on these tests, the soft tissues were interpreted as inorganic in composition^16^. Reaction of post-combustion tissue residues with hydrofluoric acid and “Kalisalz” [potassium salt] (Voigt^16^, p. 19) yielded potassium fluorosilicate crystals; this was interpreted as evidence that the preserved tissue includes a component of silicic acid^16^. Voigt suggested that preservation of the soft tissues resulted from a three-step process: (1) initial tanning of integument by humic acid (from the lake/pore waters) followed by (2) impregnation and then (3) replacement by silicic acid (“molekularer Austausch” [molecular exchange], Voigt^16^, p. 19) during diagenesis^16^. Subsequent work suggested that tanning and silicification were mutually independent and apply to different tissues^10^. The proposed source of silica is the host sediment, specifically diatoms, plant tissues rich in silica particles, and silicates^10,16^.

Voigt’s interpretation that the Geiseltal fossils are preserved, at least in part, via silicification is intriguing: this mode of soft tissue preservation usually applies to microbes, plants and arthropods^21^ but rarely to vertebrates (but see Channing, et al.^22^). Aside from the direct replacement model proposed for the Geiseltal fossils, silicification is usually considered to occur via permineralization or encrustation^21,23,24^. In each of these three mechanisms of silicification, the source of silica is universally considered to be the external environment, i.e., host sediment rich in ash and/or silica-saturated porewaters.

In sum, due to the reported high fidelity preservation coupled with replication of vertebrate soft tissues in silica, the Geiseltal biota is considered to have a unique taphonomic history. Despite this, the soft tissue taphonomy of the Geiseltal vertebrates has not been investigated in recent years. This is particularly surprising given the proposed preservation of fossil bacteria (other instances of which have been widely reinterpreted as preserved melanosomes^19,25–27^). Here, we address this issue by investigating the mode of preservation of the Geiseltal anurans using various chemical- and imaging techniques. The anurans are of particular interest because they are abundant and are reported to preserve various soft tissues with cellular-level fidelity (Supplementary Table 2)^9,10,13,28^. These include: (1) the stratum corneum and stratum germinativum of the epidermis^10,13^ plus associated bacteria^17^, (2) the stratum spongiosum and stratum compactum of the dermis, including collagen fibers^10,13^, (3) pigment cells of the stratum vasculare^10,13^; (4) muscle tissue with subcellular banding^10,13,29^, (5) connective tissue with banding^13^ and (5) statoliths of the endolymphatic sac^16^.

In this study, we assess the evidence for high fidelity preservation of the soft tissues reported by Voigt via direct replacement in authigenic silica. Our study failed to recover evidence for this mode of preservation and for preservation of most of the tissues reported originally. Claims of a unique taphonomic history and a remarkably high fidelity of preservation for the Geiseltal fossils are therefore unsupported.

## Results

For this study, we examined 168 fossil anuran specimens from the Geiseltal Collection (Natural Sciences Collections of the Martin Luther University Halle-Wittenberg; Supplementary Table 1) using light microscopy, scanning electron microscopy (SEM), energy-dispersive X-ray spectroscopy (EDS), micro-Raman spectroscopy, micro-Fourier transform infrared spectroscopy (FTIR) and electron microprobe analysis (EMPA) in order to better characterize the fidelity and mode of preservation (see Methods). One specimen is conserved on the original host sediment in water. Most of the remaining specimens are conserved on resin slabs (Supplementary Table 1)^8^, with a minority on slabs of gypsum or paraffin wax; small patches of sediment are often retained on the slabs. We analyzed 57 samples of the anuran soft tissues and 22 samples of sediment.

**Figure 1:**
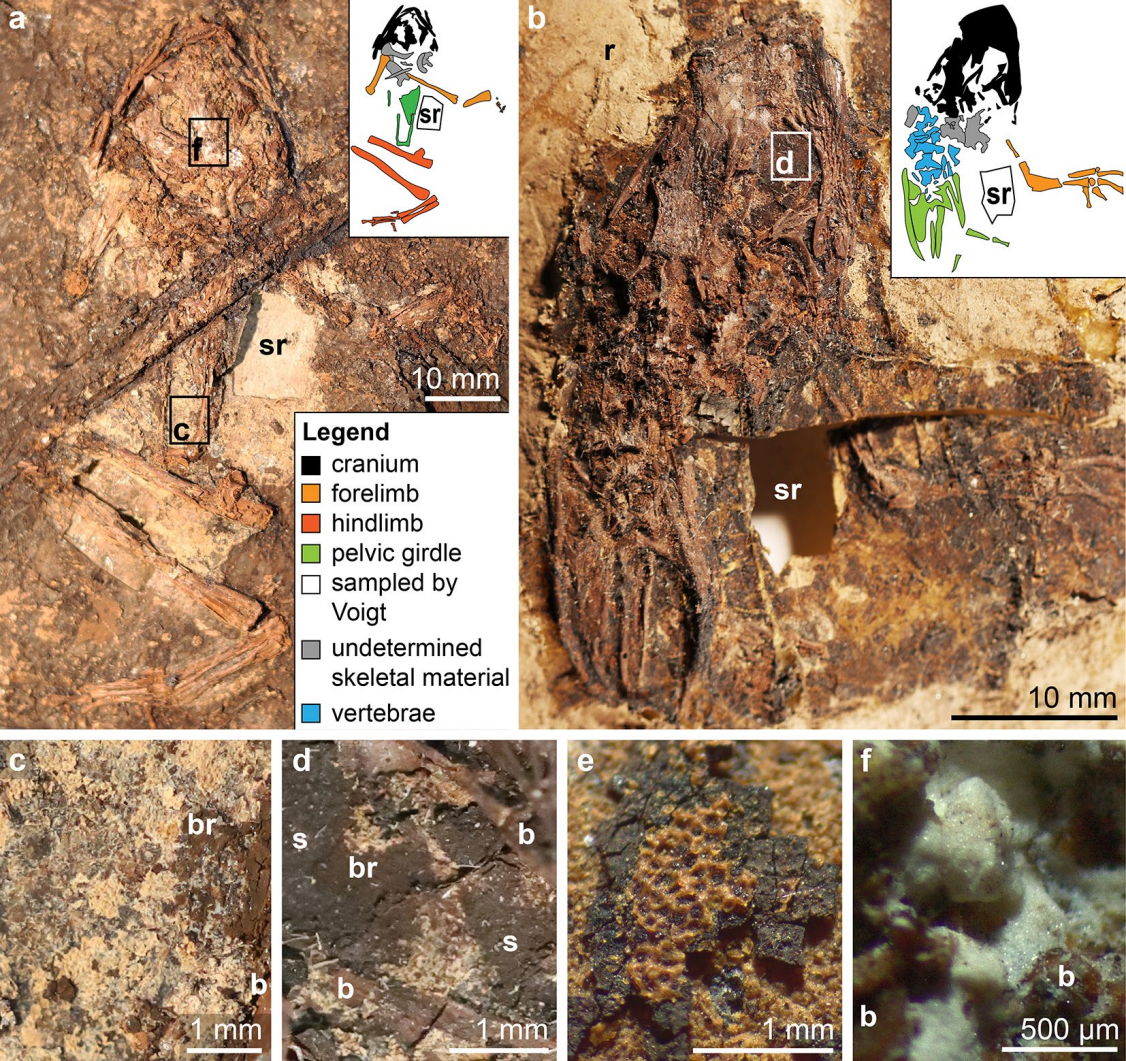
Soft tissue preservation in anurans from the Eocene Geiseltal biota. (**a**–**b**) Photographs of specimens showing extensive soft tissues, visible as a cream-colored friable material and/or a dark brown material. Boxes indicate regions shown in (**c**), (**d**) and (**f**). Insets show color-coded line drawings of the bones; Legend in (**a**) also applies to (**b**). (**a**) GMH CeIII-6698-1932 (Pelobatidae). The two parallel linear features crossing the torso are preparation artefacts. (**b**) GMH CeIII-6743-1932 (Pelobatidae). (**c**–**f**) Light micrographs showing details of the soft tissues. (**c**) Cream-colored, friable material overlain by thin brown material. (**d**) Dark grey-brown sediment (s) overlain by brown material and, in turn, the cream-colored material. Sediment and brown material are superficially similar in hand specimen but are readily distinguished using light microcopy and especially SEM. (**e**) GMH CeIII-4936a-1932 (Pelobatidae). Orange material with a striking honeycomb texture occurs in two layers, separated by a thick, blocky, dark brown material. (**f**) Pale, homogenous material in the cranium. b, bone, br, brown material, r, resin, s, sediment, sr, region sampled by Voigt.

**Figure 2:**
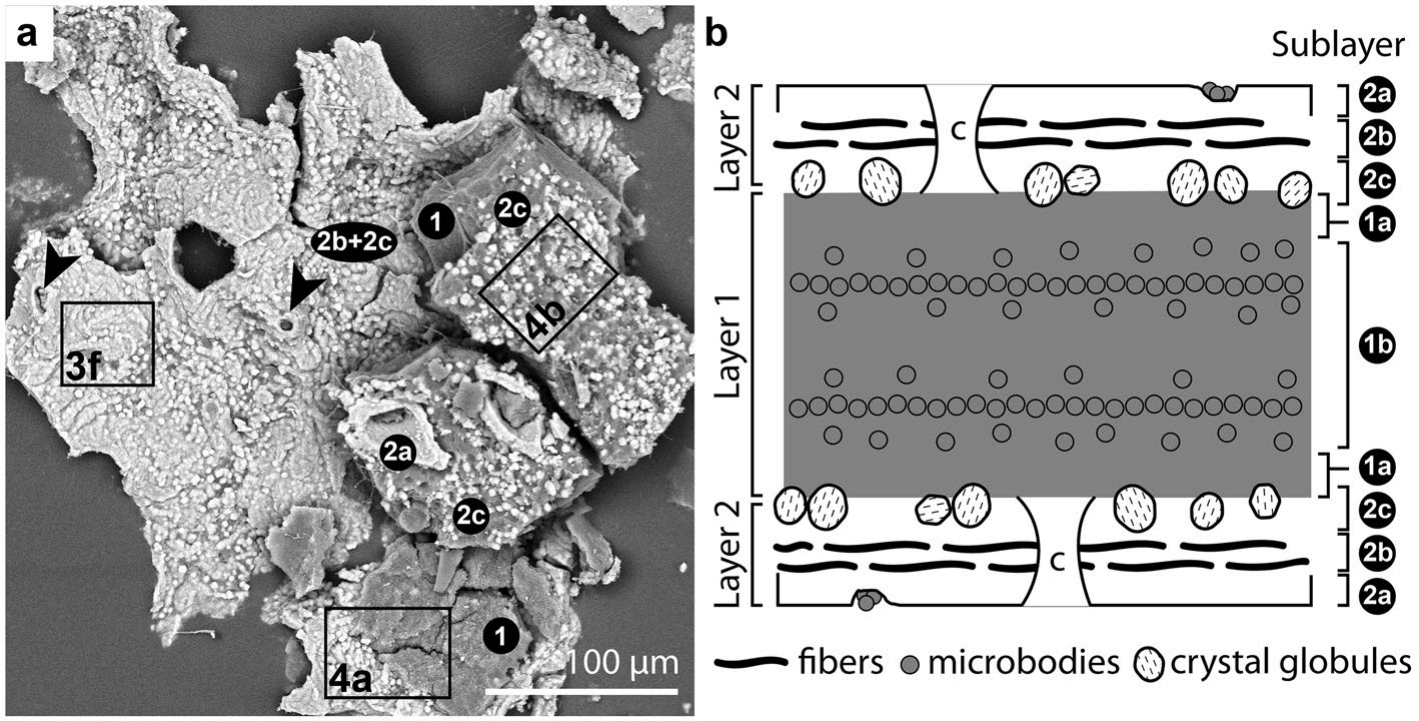
Succession of soft tissue layers in the Geiseltal anurans. (**a**) CeIII-4967-1932 (Pelobatidae). Backscatter electron image of a fractured sample of soft tissue. Not all soft tissue layers are continuous across the entire area of the sample. Bright-toned region in left center of image shows Layer 2 in ventral aspect, overlain by dark-toned Layer 1 (in right of image), which, in turn, is overlain by Layer 2. (**b**) Schematic vertical section of soft tissues. Layer 1 is sandwiched between two layers of Layer 2. Layer 2 comprises a thin, outer, amorphous sublayer (2a) underlain by a thicker fibrous sublayer (2b) and, in turn, an innermost sublayer defined by closely spaced crystal globules (2c). Layer 1 comprises an outer amorphous sublayer (1a) and an inner sublayer with zones of densely packed microbodies alternating with zones with rare microbodies (1b). c, position of centrifugal fibers.

**Figure 3:**
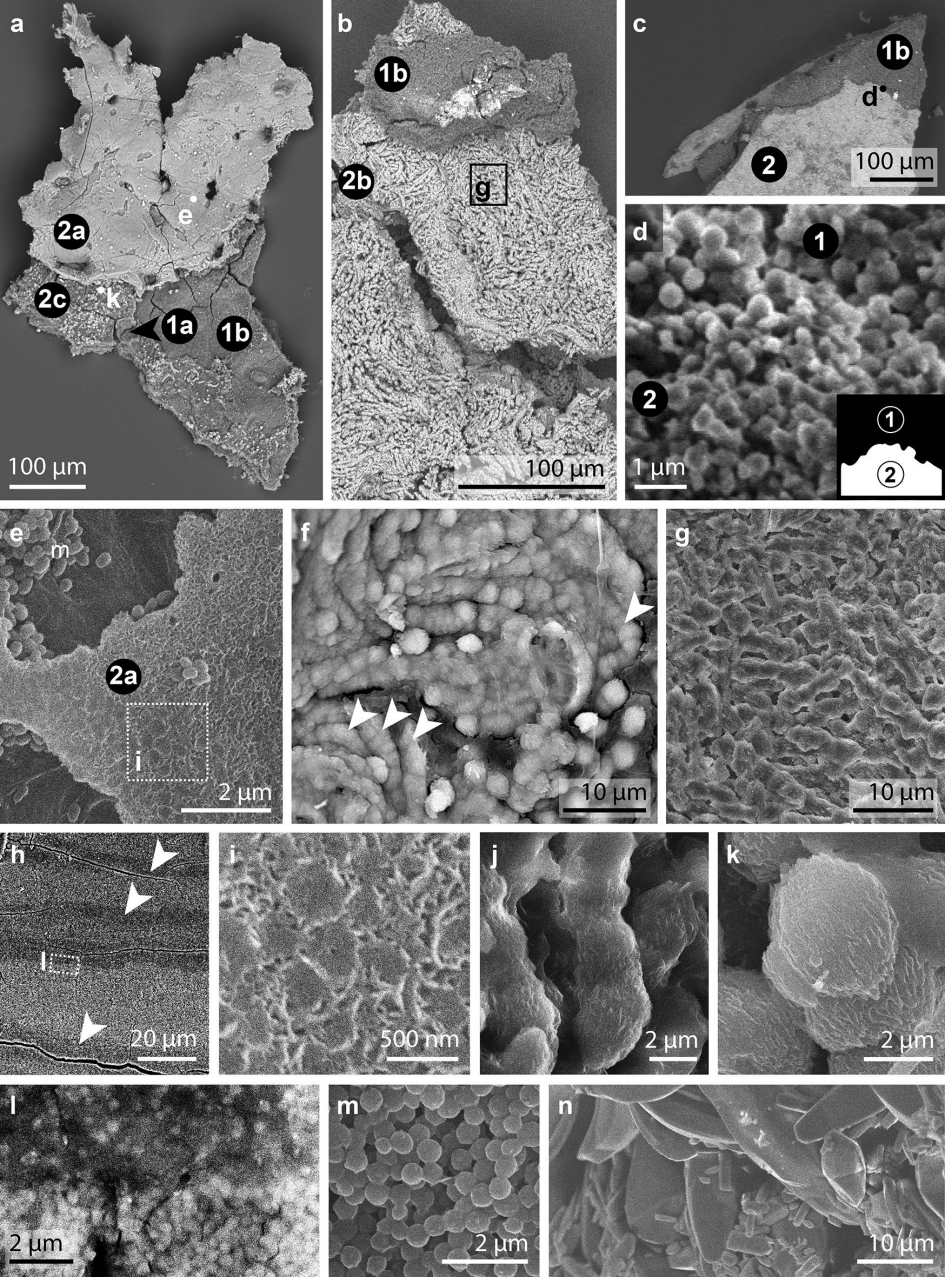
Electron micrographs of soft tissues in the Geiseltal anurans. (**a**–**c**), Backscatter electron images showing Layer 1 (dark tones) and Layer 2 (bright tones). (**d**–**n**), Secondary electron images (**d**,**e**,**i**,**j**,**k**,**m**,**n**) and backscatter electron images (**f**,**g**,**h**,**l**) showing microstructures. (**a**) Successive layers showing continuous amorphous outer surface of Layer 2 (Sublayer 2a) underlain by Sublayer 1a, in turn underlain by Sublayer 1b; Sublayers 1a and 1b show polygonal cracking. The plane of splitting has exposed Sublayer 2c locally where Sublayers 2a and 2b separated into the counterpart. (**b**) Inner surface of Layer 2 showing curved to sinuous fibers of Sublayer 2b, overlain by Sublayer 1b (top of image). (**c**) Layer 2 underlain by Layer 1. (**d**) Detail of region indicated in c showing Layer 2 underlain by Layer 1. Layer 2 comprises small irregular granules that are often juxtaposed or conjoined. Layer 1 comprises round microbodies. (**e**) Detail of region indicated in (**a**), showing outer surface of Layer 2 with distinctive dimpled texture; see (**i**) for detail. Microbodies occur on the layer surface and in surface cavities. (**f**) Sinuous fibers of Layer 2 (Sublayer 2b) crudely defined by irregular globules of calcium phosphate (see **j** and **k**). (**g**) Detail of region indicated in (**b**). (**h**) Backscatter electron micrograph showing alternating microbody-poor (arrows) and microbody-rich sublayers in Layer 1 in the torso. (**i**) Detail of region indicated in e showing dimpled outer surface of Layer 2. (**j**) Detail of the fibers in Sublayer 2b showing closely spaced to juxtaposed, irregular, poorly defined globules with similar nanotexture to those in Sublayer 2c. (**k**) Close-up of individual globule from Sublayer 2c showing irregular surface and margins, with a poorly defined fibrillar nanotexture. (**l**), Detail of (**h**). Transition of microbody-rich to -poor sublayer in Layer 1. (**m**) Microbodies from Layer 1 in the torso. (**n**) Densely packed euhedral crystals comprising pale material in cranium. (**a**,**e**,**f**,**i**,**k**,**m**) GMH CeIII-4967-1932; (**c**,**b**,**d**,**g**,**n**) GMH CeIII-6698-1932; (**h**,**j**,**l**), GMH CeIII-4936a-1932.

Light microscopy and SEM imaging reveal that the soft tissues of the Geiseltal anurans are preserved as three materials that differ in their spatial distribution, color, texture, thickness, and chemical characteristics (Fig. 1). Two materials are layered and are referred to here as Layers 1 and 2. Due to variations in the plane of splitting among and within individual specimens, each layer may not be visible over the full extent of the soft tissues in each specimen (Figs. 1, 2 and 3a–c); the third material occurs only in the posterior of the cranium and along the vertebral column (Fig. 1a,f).

Layer 1 occurs in 90% (total n = 168) of specimens (100% of palaeobatrachids (total n = 15) and 93% of pelobatids (total n = 54)). Within an individual specimen, it can occur in some, or all, of the following body regions: eyespots, torso, abdomen and limbs (Fig. 1a–e). The layer is usually black to brown in color, with a microgranular texture and polygonal cracking (Figs. 1e, 2a, 3a; Supplementary Fig. 1). It can extend over an area of several square centimeters and is over- and underlain by Layer 2 (Figs. 1e and 2a). Layer 1 usually comprises two sublayers. The outermost sublayer (1a) is amorphous and ca. 10–20 µm thick (Fig. 2b; Supplementary Fig. 1). This outer amorphous sublayer transitions gradually to an inner sublayer (1b) rich in microbodies, each ca. 0.5–3 µm long (Fig. 3m; Supplementary Fig. 1c,d,g). Sublayer 1b comprises several bands of densely packed microbodies that alternate with bands dominated by amorphous matrix (and with few microbodies; Fig. 3h,l; Supplementary Fig. 1). In several samples, Layer 1 lacks Sublayer 1a, comprising densely packed microbodies (Sublayer 1b) only. The microbodies lack a preferred orientation.

EDS spectra of Layer 1 show peaks for carbon, oxygen and sulfur (Supplementary Figs. 1 and 3). EPMA reveals a composition that includes sulfur, calcium and traces of oxygen, phosphorus, fluorine, and chlorine (Supplementary Table 3); these data confirm that Layer 1 is organic in character.

Layer 2 occurs in 70% of specimens (67% of palaeobatrachids and 83% of pelobatids). Within an individual specimen, it can occur in some, or all, of the following body regions: cranium (including eyespots), torso, abdomen and hindlimbs. It is 20–30 µm thick (Fig. 3h; Supplementary Fig. 2) and its preservation shows a spectrum between two end-member states. In 11% of specimens, Layer 2 is exclusively yellow to orange in color (Fig. 1b), cohesive, continuous and brittle, with a striking honeycomb texture (Fig. 1e); it can extend over an area of up to several square centimeters. The honeycomb texture includes both depressions in the surface of the layer plus perforations that expose the underlying Layer 1 (Fig. 1e; Supplementary Fig. 2). In 30% of specimens, Layer 2 is exclusively pale yellow, friable and discontinuous, with an amorphous texture (Figs. 1a–d, 3c; Supplementary Fig. 2). In 29% (n = 168) of specimens, both states of Layer 2 are present (Fig. 1a,b).

**Figure 4:**
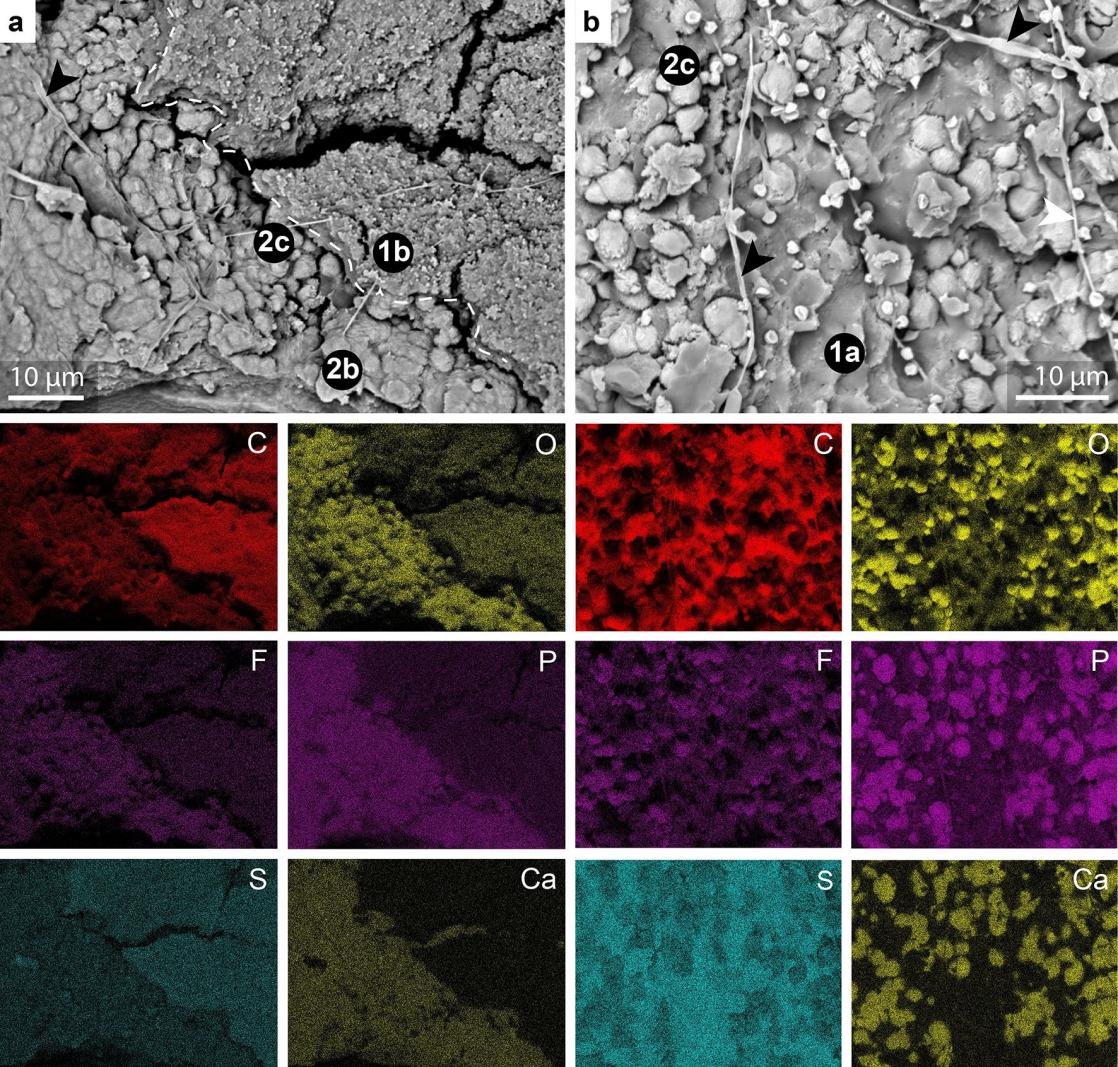
Chemical mapping of the soft tissues of GMH CeIII-4967-1932; see Methods for analytical parameters. (**a**–**b**) Backscatter electron images showing Layers 1 and 2. (**a**) shows Sublayers 1b, 2b and 2c. (**b**) shows Sublayers 1a and 2c. Elongate features on the sample surface are interpreted as fungal contaminants (arrowheads). (**a**) Sublayers 2b and 2c are enriched in Ca, P and O and, to a lesser extent, F. Sublayer 1b is enriched in C and S. (**b**) Irregular globules of Sublayer 2c (see also Fig. 3k) are enriched in Ca, P and O and, to a lesser extent, F. Sublayer 1a is enriched in C and S.

Where Layer 2 shows a cohesive texture, SEM analysis reveals three sublayers that can be discriminated on the basis of their texture. The outermost sublayer (2a) shows a pattern of perforations that expose the underlying Layer 1; the perforations are oval to irregular in shape and ca. 10–30 µm wide, with a spacing of 100–200 µm (Fig. 3a). High-magnification images of the external-facing surface of the layer reveal a network of oval to irregular-shaped microdepressions, each ca. 0.5 µm wide (Fig. 3e,i). The outer surface of the layer often shows clusters of spheroidal to ovoid microbodies, each ca. 0.5 µm long (Fig. 3e). The inner-facing surface of Layer 2 reveals Sublayers 2b and 2c (Fig. 3b,f,g). Sublayer 2b comprises densely packed fibers (each ca. 5–10 µm long and 2–3 µm wide) that are orientated (sub-)parallel to the layer surface (Figs. 2a, 3b,g,j; Supplementary Figs. 1 and 2); some fibers are curved to sinuous (Fig. 3b,f), especially adjacent to the perforations that penetrate the layer. Each fiber comprises a chain of juxtaposed, poorly defined, globules each ca. 2–3 µm long (Figs. 2a and 3f,j); each globule has a distinctive surficial microfabric formed by aligned ridges and grooves (Fig. 3j). This microfabric usually shows a consistent orientation in adjacent globules within a single fiber (Fig. 3j). Sublayer 2b transitions gradually to Sublayer 2c, the innermost sublayer (Figs. 2a and 3a,f,k). The latter is characterized by dispersed globules similar in shape, size and texture to those in Sublayer 2b (Figs. 3k and 4b). Where the plane of splitting removes the globules into the counterpart, the surface of the underlying Layer 1 shows external molds of the globules (Fig. 4b).

**Figure 5:**
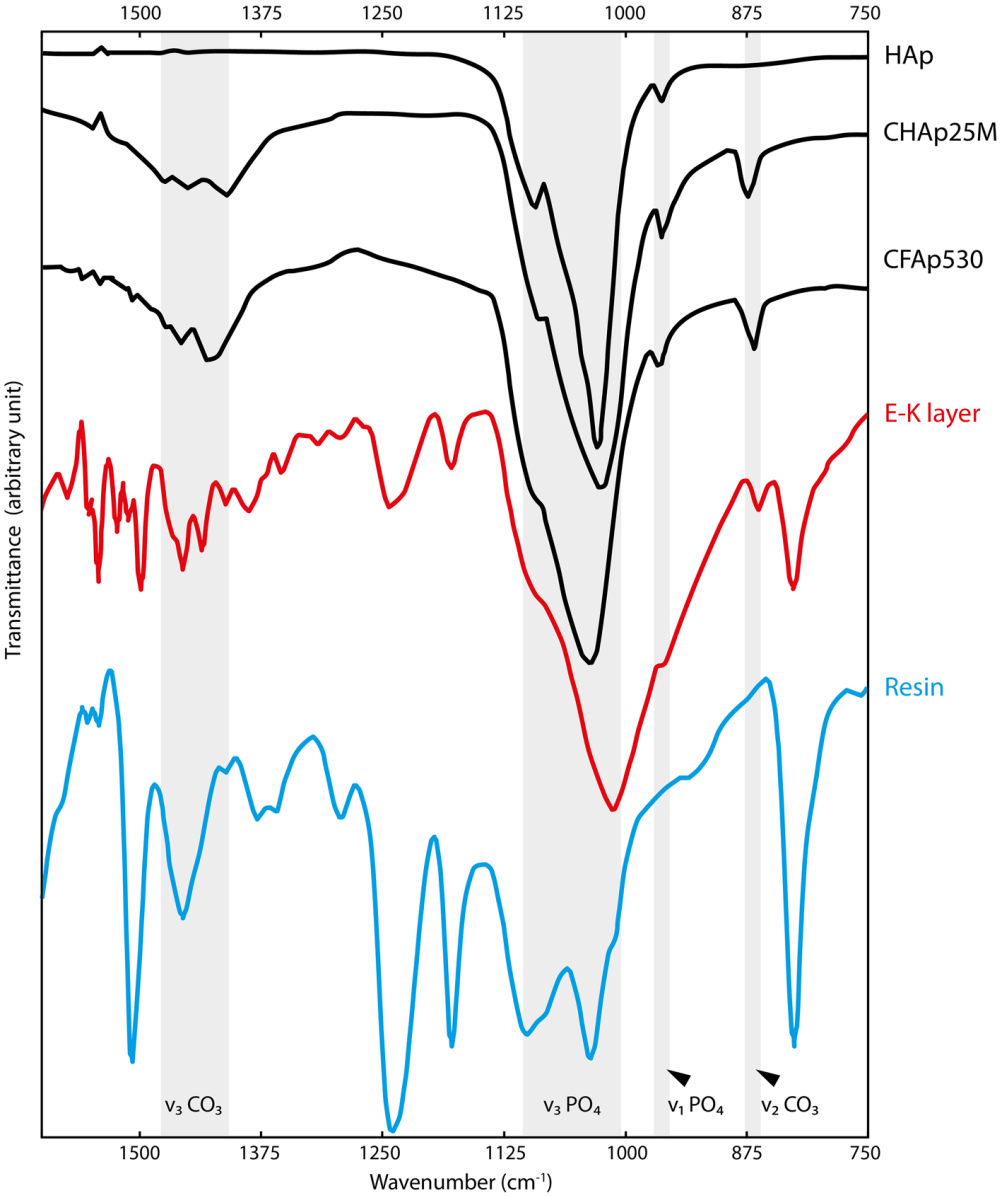
FTIR spectroscopy of the mineral phase that replicates the E–K Layer (GMH CeIII-4936a-1932; Pelobatidae). The FTIR spectrum of the E–K layer (red) shares several bands with spectra of carbonated hydroxyapatite (CHAp25M) and carbonated fluorhydroxyapatite (CFAp530). The peaks at 1509 cm^−1^, 1242 cm^−1^, 1180 cm^−1^, and 828 cm^−1^ probably derive from the embedding resin (modified from Antonakos et al.^30^).

EDS spectra from Layer 2 show peaks for calcium, phosphorus, oxygen and fluorine (Supplementary Figs. 1 and 3); the composition of the layer is thus interpreted as calcium phosphate, with a likely (albeit minor) component of fluorapatite. This composition is supported by EPMA (Supplementary Table 3), which reveals that Layer 2 has a composition consistent with apatite. In samples where Layer 1 and Layer 2 co-occur, EDS maps confirm the contrasting composition of Layer 1 and Layer 2 (Fig. 4a,b; Supplementary Fig. 1). FTIR spectra reveal peaks centered at 963 cm^−1^ (v_1_PO_4_), 1012 cm^−1^ (v_3_PO_4_) and 1456 cm^−1^ (v_3_CO_3_), consistent with carbonated hydroxyapatite (Fig. 5; Supplementary Table 4)^30–32^. Other peaks centered at 864 cm^−1^ (v_2_CO_3_), 1092 cm^−1^ (v_3_PO_4_), 1436 cm^−1^ and 1456 cm^−1^ (v_3_CO_3_; Fig. 5; Supplementary Table 4) are characteristic of carbonated fluorapatite (francolite)^30–32^. The material preserving Layer 2 therefore probably represents a mixture of both mineral phases. Notably, the v_2_CO_3_ band indicates a carbonate ion substitution for the phosphate tetrahedron (B-type substitution)^30^, which suggests a biological apatite^30^.

In 42% of specimens (47% of palaeobatrachids and 63% of pelobatids), a white material occurs in the posterior part of the cranium and along the vertebral column (Fig. 1a,f). SEM analysis reveals densely packed, short euhedral crystals (Fig. 3n; Supplementary Fig. 3). Individual crystals are ca. 1–30 µm long; some show evidence for multiple phases of growth (Supplementary Fig. 3). EDS spectra reveal peaks for calcium, oxygen, and carbon, consistent with calcium carbonate (Supplementary Fig. 3). This composition is supported by Raman spectra, which reveal a major peak at 1084 cm^−1^ (v_1_CO_3_^2−^) and minor peaks at 152 cm^−1^, 179 cm^−1^, 205 cm^−1^ and 704 cm^−1^ (v_4_CO_3_^2−^; Supplementary Fig. 3); this spectrum is characteristic of aragonite^33^.

Differences between taxonomic groups in the number of specimens that show Layer 1, Layer 2 and the white material are not statistically significant (independent samples t-test, *p* = 0.664) and may reflect differences in sample size.

The vast majority of fossil-bearing sediments at Geiseltal have been previously characterized as “Weichbraunkohle” [soft brown coal] (Pickel and Wolf^34^, p. 482). Our SEM, EDS, Raman and XRD data (for the sediment associated with the anurans) confirm a composition dominated by organic matter with almost no detrital minerals such silicates or clays (Supplementary Note; Supplementary Fig. 5, Supplementary Table 5).

## Discussion

The microstructures preserved in the soft tissues of the Geiseltal anurans can be interpreted in the context of soft tissue microstructures preserved in fossil vertebrates from other Konservat-Lagerstätten. The microbodies of Layer 1 resemble microstructures associated with other fossil vertebrates that were initially interpreted as fossil bacteria^17,35–37^. In recent years, however, microbodies with a similar geometry and spatial distribution associated with diverse fossil vertebrates have been interpreted as fossil melanosomes^19,38–42^; these interpretations are supported by extensive chemical evidence for fossil melanin^18,26,38,42,43^. Even though the melanosome hypothesis is now widely accepted, it is nonetheless important to investigate new fossil reports from first principles^44^, as in the text that follows here.

The size and geometry of the fossil microbodies in the Geiseltal anurans (ca. 0.6 ± 0.4 µm long; Fig. 3e,m; Supplementary Fig. 1) are consistent with those of melanosomes preserved in other fossil anurans (ca. 0.6 µm long^42^; ca. 0.4–1.2 µm long^19,40^) and in extant anurans (ca. 0.2–1.5 µm long^42^), but also with modern bacteria (0.2–5 µm^45^). Interpretations of the nature of the fossil microbodies should therefore consider additional attributes, such as the spatial distribution of the microbodies, as follows.

In decaying carcasses, bacteria can form external biofilms (derived from the integument and/or sediment) and internal biofilms (derived from the gut flora) that degrade soft tissues^46,47^. The external surface of Layer 2a (the outermost of the preserved soft tissue layers in the fossils), however, preserves only rare microbodies (Fig. 3e); most of the fossil microbodies are sandwiched between the two layers of Layer 2. This distribution of microbodies is not consistent with bacterial overgrowth of carcasses. Further, the stacking of successive microbody layers in Layer 1, where microbodies have size-specific geometries in each layer (Supplementary Fig. 1), is difficult to explain as a bacterially generated structure. Instead, this feature is consistent with a melanosome origin^20,44^: melanosomes in extant and fossil vertebrates have tissue-specific geometries^42^. Given that melanosomes are resistant to degradation^20,48^, collapse of the soft tissues during decay and extended degradation of body tissues would therefore ultimately yield melanosome-rich tissue residues^20^. Size-specific layering can be generated where melanosomes from different tissues are superimposed, and where carcasses are not disturbed by bottom currents during exposure on the sediment surface^20^. In the Geiseltal anurans, the layering of microbodies from different populations (defined by geometry) is consistent with decay-induced layering of melanosomes derived from different tissues^42^.

Collectively, these data indicate that the fossil microbodies are extremely unlikely to represent fossil bacteria; the microbodies are most parsimoniously interpreted as fossil melanosomes.

Several notable features of the Geiseltal melanosomes are treated briefly here. The melanosomes lack distinctive surface nanotextures, as reported in some modern melanosomes^19,37,49^ and melanosomes from the Libros biota (Miocene, Spain)^18,19^. This may reflect a taxonomic and/or tissue-specific signal, enrichment in pheomelanin^37^ or a low fidelity of preservation (taphonomic bias). Unlike the Libros anuran melanosomes, some of which are partially replaced in calcium phosphate^19^, those in the Geiseltal anurans show no calcium phosphate mineralization. This likely reflects taphonomic differences between the two biotas, e.g., the availability of phosphate ions and the timing of phosphatization (see below). The layers of amorphous, melanosome-poor material that separate layers of melanosomes in the soft tissues (Fig. 3h,l; Supplementary Fig. 1) are difficult to interpret conclusively. The amorphous layers may represent residues of tissues that had limited, or no, melanin in vivo*.* Alternatively, they may represent remains of degraded melanosomes^50^.

The melanosomes of Layer 1 are rich in sulfur; this is consistent with the chemistry of melanosomes from other fossil biotas including Libros (Miocene, Spain^19^), Bolca (Eocene, Italy^39^) and Messel (Eocene, Germany^51^) and suggests preservation via sulfurization^19^. This process involves diagenetic incorporation of bacterially- or decay-generated sulfides (and/or polysulfides) into organic matter under anoxic conditions^52^. Sulfurized tissues are highly cross-linked and thus resistant to bacterial degradation^53^. Sulfurization is common in dysoxic to euxinic, organic-rich sediments deposited in restricted basins with a stratified water column and abundant bacterial sulfate reduction in the upper part of the sediment column^19^. In such settings, the source of sulfur is considered to be the organic-rich host sediment^19^. Preservation of the Geiseltal melanosomes via sulfurization is supported by the preservation of fossil latex (monkeyhair laticifers) in the laminated sediments^54^; this material is formed by natural low-temperature sulfurization of plant tissue at diagenetic temperatures of < 100 °C^55^.

**Figure 6:**
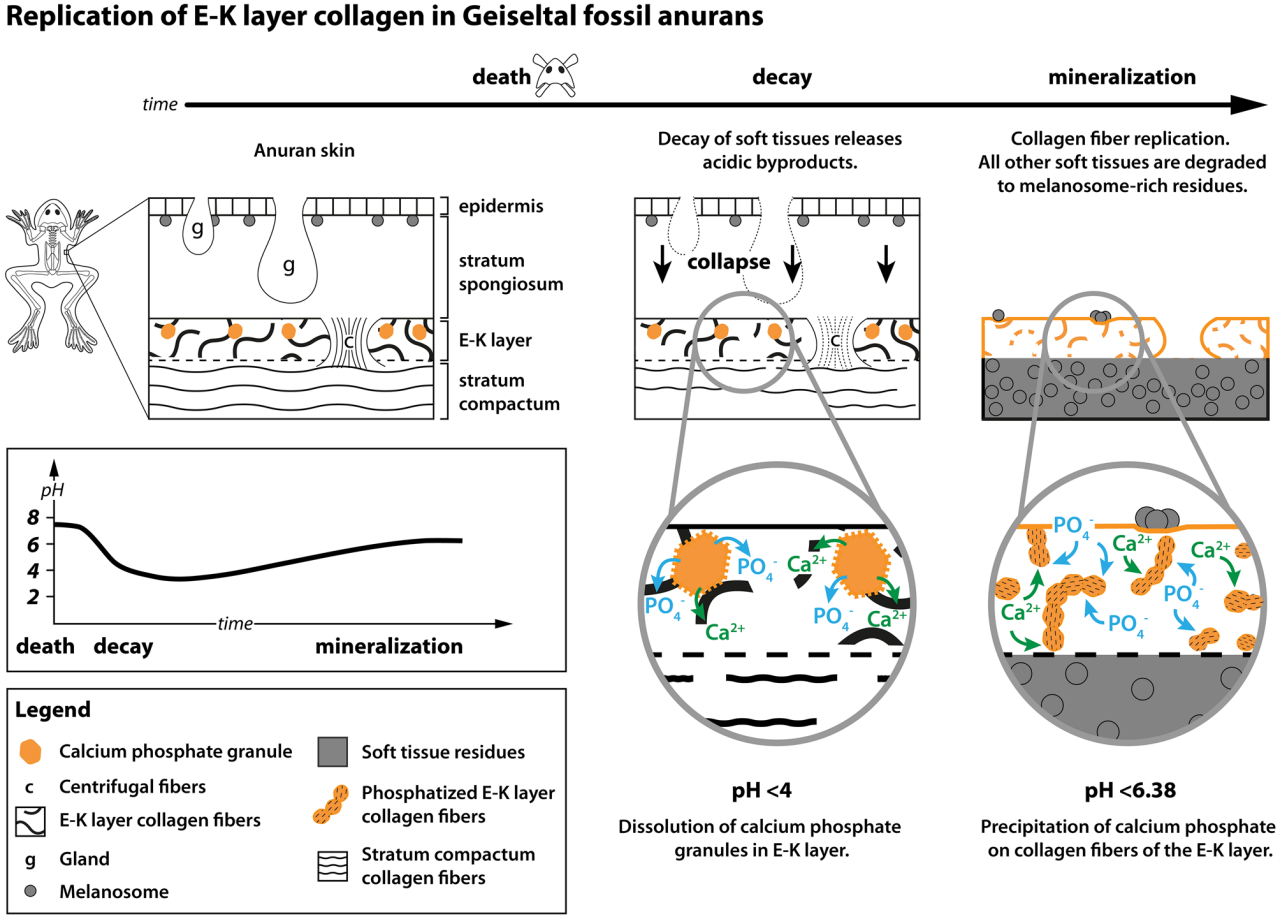
Taphonomic model for the preservation of the E–K layer in the Geiseltal anurans.

Layer 2 is interpreted as the mid-dermal Eberth–Katschenko (E–K) layer of anuran skin. In extant anurans, the E–K layer comprises collagen fibers, glycosaminoglycans, proteoglycans and calcium phosphate granules^56–59^. The layer is situated between the stratum spongiosum (of the outer dermis) and stratum compactum (of the inner dermis; Fig. 6). Centrifugal fibers perforate and traverse the E–K layer vertically, connecting the overlying stratum spongiosum with the underlying stratum compactum and hypodermis^56,57^. The columns form a regular pattern (spaced ca. 100–500 µm apart)^56,57,60^. As a result, the E–K layer in extant anurans shows a honeycomb pattern in plan view.

Layer 2 is interpreted as the preserved remains of the E–K layer based on the following features: (1) both the fossil layer and the E–K layer have a sharp, well-defined outer surface; (2) the irregular, poorly-defined inner surface of the fossil layer is consistent with the E–K layer in extant anurans, where collagen fibers of the layer interweave with those of the underlying stratum compactum^56,57^; (3) the fossil layer is ca. 3–30 µm thick (Fig. 3h); this range of thicknesses is consistent with that of the E–K layer in extant anurans (1–30 µm^57,58,61^); (4) The presence of two successive layers of Layer 2 in the fossils (Fig. 2a) is consistent with the superposition of the E–K layer from the dorsal and ventral skin during decay and collapse of the carcass; and (5) the size of the perforations and the perforation pattern (Fig. 1e; Supplementary Fig. 2) that form the honeycomb texture in the fossils are consistent with those of the centrifugal fibers in extant anurans (3–4 per 100 µm^2^ and 10–50 µm in diameter in extant anurans)^56,57,60^.

All of the above features also characterize the preserved E–K layer in fossil anurans from the Libros biota^18^; in addition, the collagen fibers preserved in fossils from both localities are usually sinuous where continuous. There are, however, a number of notable differences between the preserved features of the E–K layer in the Geiseltal anurans, those preserved in the Libros frogs and those described in extant anurans. First, the constituent fibers of the E–K layer are thicker (ca. 2–4 µm thick; Fig. 3f,g,j) in the Geiseltal anurans than in the Libros and extant frogs. In extant frogs, the collagen fibers of the E–K layer are each ca. 0.1–2 µm thick^56,57,60^; the fibers in the Libros frogs are ca. 1–2 µm thick^18^ and were thus interpreted as preserved individual collagen fibers. The fibers in the Geiseltal anurans are shorter (ca. 10–20 µm; Fig. 3b,g) than those in the Libros frogs (ca. 20–100 µm^18^). Despite these differences, the Geiseltal fibers are considered to be best interpreted as individual collagen fibers and not fiber bundles; the latter would be expected to vary more in width than the preserved fibers.

The preserved E–K layer is replicated in calcium phosphate (Fig. 4a); no other soft tissues in the Geiseltal anurans share this mode of preservation. Phosphatization of soft tissues is controlled, at least in part, by the availability of phosphate ions (PO_4_^3−^)^62–64^ and thus the source(s) of phosphate ions must be considered. Selective preservation of the skin, the most surficial tissue of the body, would be consistent with (a) sedimentary source(s) of phosphate ions, e.g., sedimentary organic matter, biogenic apatite, benthic microbial mats and redox cycling of phosphorus by iron oxyhydroxides^65^. The mode of conservation of the specimens renders it difficult to completely exclude these potential sedimentary sources, as only limited volumes of sediment are available for analysis. Based on the sediment analyzed in this study, however, there is no evidence for sedimentary apatite, benthic microbial mats, iron minerals or abundant phosphate minerals (Supplementary Fig. 5 and Table 5); there is thus no obvious sedimentary source of phosphate.

The fine lamination of the sediment (Supplementary Fig. 4) suggests low sedimentation rates and extended exposure (weeks to months) of the specimens on the lake floor without being completely covered by sediment. If sedimentary organic matter were the primary phosphate source^66^, specimens might therefore be expected to show preferential phosphatization of skin on only one side of the body (i.e., the surface in contact with the sediment)^65^. Further, this phosphate source might also be expected to result in the preservation of the epidermis and upper dermis (assuming that the outer skin layers have a similar propensity to bind calcium as the E–K layer collagen). Neither of these features, however, are present in the Geiseltal anurans.

In sum, the preservation of both the ventral and dorsal E–K layer, the restriction of phosphatization to this specific component of the skin and the lack of evidence for sedimentary phosphate are inconsistent with a sedimentary source of phosphate and instead collectively indicate an internal phosphate source, i.e., from inside the decaying carcass. Bones are an obvious phosphate source but show no evidence for dissolution; the phosphate must therefore have derived from the soft tissues.

Collagen is decay-resistant and readily promotes nucleation of calcium phosphate^18,62,67^. The E–K layer is rich in calcium phosphate granules in vivo^56,57,68^ and thus contains a local phosphate source for the replication of the collagen fibers of the E–K layer (Fig. 6). The decreasing fidelity of fiber preservation from the external part of the E–K layer towards more internal zones is consistent with higher concentrations of calcium phosphate granules in the outer regions of the E–K layer, as in extant anurans^57,68^. The absence of other preserved soft tissues may reflect a lack of internal phosphate sources and/or the complete degradation of other tissues prior to phosphatization (possibly due to proximity to the source of decay bacteria). The skeletal taphonomy of the Geiseltal anurans indicates prolonged transport in surface waters and/or prolonged exposure on the lake floor^8^. This, plus warm surface water temperatures^8^ may have facilitated (partial) decay of soft tissues, including connective tissues and internal organs, prior to deposition. The skin, however, is likely to have retained cohesion until late decay^69^.

In our taphonomic model (Fig. 6), calcium and phosphate ions originated from the dissolution of E–K layer calcium phosphate granules during late decay^18^. This required pH < 4^70,71^. Such conditions may exist, even in a highly localized fashion (i.e., as microenvironments^18^), in a carcass following the liberation of acidic byproducts of decay^72^. Rapid dissolution of the granules would have generated local high concentrations of phosphate. Reprecipitation of calcium phosphate (i.e., apatite) on collagen fibers of the E–K layer likely required a slightly higher pH value, but < pH 6.38 (i.e., below the carbonic acid dissociation constant) and where concentrations of dissolved phosphate in the tissue were sufficiently high to inhibit calcium carbonate precipitation^73^. The large crystals of calcium phosphate and associated low fidelity of preservation of the collagen fibers (Fig. 3j,k) likely reflects two factors: extended decay of the collagen fibers (yielding a low density of suitable nucleation points for calcium phosphate but generates space between nucleation points for increased crystal sizes) and local high phosphate concentrations, thus favoring crystal growth over nucleation. Continued growth and ultimately merging of adjacent crystals would have crudely replicated the (sinuous) morphology of the original fibers (Fig. 3f). Individual globules and short lengths of merged globules (Fig. 3k) in the lower E–K layer (Sublayer 2c) likely reflect lower concentrations, or later availability, of calcium phosphate during decay, precluding replication of entire fibers. Aligned nanotextures in adjacent globules (Fig. 3k) may reflect an underlying biomolecular/stereochemical template.

The epidermis, the upper dermis, and the centrifugal fibers (Fig. 2b) of the skin are not preserved. This suggests that the various cellular and non-cellular components of those soft tissues have a lower capacity to bind calcium than the E–K layer fibers and/or decayed completely before the tissue entered the phosphatization window.

In summary, our model for phosphatization of the E–K layer of the Geiseltal anurans comprises the following steps (Fig. 6): (1) extensive decay of the carcasses led to liquefaction of internal tissues, but only partial decay of collagen in the E–K layer. Other skin components may have been present. (2) The release of acidic decay products locally lowered pH to < 4 and dissolved the granules of the E–K layer. (3) Calcium and phosphate ions diffused the short distance to the E–K layer collagen fibers. (4) Calcium phosphate precipitated where pH < 6.38 and the affinity of the collagen to bind calcium ions was high. (5) Any other remaining skin components decayed completely because they had a lower affinity for phosphate and/or the available phosphate was consumed by replication of the E–K layer collagen.

The location and size of the aragonite crystals in the cranium and along the vertebral column are consistent with those of crystals of the endolymphatic sac. In extant anurans, this organ encircles the hindbrain and forms cysts between each pair of successive vertebrae^74,75^; it stores and mobilizes calcium for skeletal development^74–76^. The sac contains several generations of aragonite crystals^74,76^ that are similar in size (1–10 µm) and habit (euhedral, oval or elongated with broad peaks on both sides; Fig. 3n)^74,76^ to the crystals in the Geiseltal anurans (Fig. 3n). The masses of aragonite in the Geiseltal anurans are therefore likely to represent original crystals of the endolymphatic sac. This suggests local pH values > 6.38 for the duration of decay^72,73^. This decay microenvironment may have been promoted by the presence of bounding membranes during the early stages of decay (when most organic acids are liberated). In extant anurans the size of the endolymphatic reserves varies among families^77^ and, within a single species (e.g., metamorphosing tadpoles of *Rana temporaria*), larger endolymphatic reserves are associated with high environmental calcium concentrations^75^. This suggests that Geiseltal anurans with particularly large volumes of aragonite crystals lived during periods where the lake waters were relatively rich in calcium.

Our analysis of the proportions of specimens that show the melanosome layer, the E–K layer and aragonite crystals—in particular, the absence of significant differences between palaeobatrachids and pelobatids—suggests that taxonomy does not impact preservation.

In both the Libros and Geiseltal anurans, the E–K-layer is replicated in calcium phosphate and envelopes melanosome-rich carbonaceous layers that are preserved via sulfurization. The fidelity of preservation of the E–K layer, however, is higher for Libros specimens; further, the melanosomes of Libros specimens are partially mineralized^19^. The higher fidelity of the Libros E–K layer collagen fibers relative to the Geiseltal specimens may reflect less decay prior to mineralization as (1) more intact collagen fibers would have more nucleation sites, thus limiting crystal growth and (2) lower concentrations of decay acids. For the Libros anurans, both elements would have constrained the growth of calcium phosphate crystals, facilitating finer replication of fibers than at Geiseltal. By the time the skin of the Geiseltal anurans entered the phosphatization window, it may not have been sufficiently intact to allow high fidelity replication.

Another contributing factor to this taphonomic variation may be the amount of phosphate ions present in the E–K layer in vivo: the layer varies in thickness and lateral extent among extant anurans^57,61^.

In conclusion, our reinvestigation of the fossilized soft tissues of the Geiseltal anurans failed to find evidence for many tissue components reported formerly, i.e., bacteria, blood vessels, cellular details of the epidermis, collagen of the stratum spongiosum and stratum compactum, muscles, and pigment cells. Compounding this, there is no evidence for the preservation of soft tissues via silicification. We can, however, confirm the preservation of certain tissue- and ultrastructural features: melanosomes (preserved via sulfurization), collagen fibers of the mid-dermal E–K layer (replicated in calcium phosphate) and the aragonite crystals of the endolymphatic sac. Claims of a unique taphonomic history and an unusually high fidelity of preservation for the Geiseltal fossils are therefore unsupported, requiring reevaluation of the capacity of the fossil record to preserve labile vertebrate soft tissues as three-dimensional silica replacements. Instead, the Geiseltal biota highlights an emerging taphonomic pattern for fossil vertebrates in lacustrine ecosystems, i.e., body outlines defined (in whole or part) as melanosome films and localized mineralization of tissues in calcium phosphate, where microenvironments rich in phosphate develop during decay and where tissues have a high potential for phosphatization. This taphonomic pattern applies to biotas such as Las Hoyas (Cretaceous, Spain)^78,79^, Ribesalbes (Miocene, Spain)^80^, Libros (Miocene, Spain)^18,19^ and Bechlejovice (Oligocene, Czech Republic)^80^. Our results highlight the importance of the reevaluation of historic fossil collections using modern techniques in order to unveil incorrect interpretations and scrutinize established scientific paradigms.

## Methods

### Fossil material

The middle Eocene Geiseltal locality is located ca. 20 km southwest of Halle (Saale) in Saxony-Anhalt, Germany. The geology of the site is reviewed in detail in Falk et al.^8^ and a summary is provided here. In brief, the vast majority of Geiseltal fossil vertebrates are hosted within lignites, which occur at various intervals in a ca. 120 m thick succession of palustrine, limnic-palustrine and fluvial sediments^9,81,82^. The lignites were commercially exploited in a series of open cast mines for ca. 100 years and most anuran specimens were recovered during the 1930s^28,83^. The mines were flooded in 2003 and the fossiliferous sites are no longer accessible.

The Geiseltal Collection is housed in the Natural Sciences Collections (ZNS) of the Martin Luther University Halle-Wittenberg in Halle (Saale; Saxony-Anhalt, Germany). Most of the fossil anurans are conserved via the transfer method, whereby the bones and soft tissues (and sometimes patches of associated host sediment) were transferred to nitrocellulose glue or wax in order to facilitate long-term storage (see Falk et al.^8^ for details).

For this study, we examined 168 fossil anuran specimens from the Geiseltal Collection. The dataset (Supplementary Table 1) is identical as that used in Falk et al.^8^, comprising Pelobatidae (n = 54; which are predominantly terrestrial), Palaeobatrachidae (n = 15; which are predominantly aquatic) and anurans of undetermined affinity (n = 99). Some specimens may represent Discoglossidae^84,85^. All specimens represent post-tadpole developmental stages, but discrimination of adults, subadults and juveniles was not possible.

Specimens are usually well-articulated but vary in completeness^8^. Most specimens are conserved in nitrocellulose glue (Fig. 1a,b), with a minority in paraffin wax. One specimen is in situ in the original sedimentary matrix; the slab is conserved in water. Many specimens are associated with patches of dark organic-rich sediment (Fig. 1d). The distinction between this sedimentary matrix and potential soft tissue, however, is not always clear; it was not, therefore, assumed a priori that patches of dark material adjacent to the bones represent preserved soft tissue. Most slabs show residues of pale grey clay at and close to the slab margins; these residues derive from the resin transfer process^8,86^. Some specimens are associated with plant remains (n = 33).

### Scanning electron microscopy (SEM) and energy-dispersive X-ray spectroscopy (EDS) analysis

Small (1–2 mm^2^) fragments of soft tissues were dissected from six specimens using sterile tools. Samples of sediment (dark material from regions beyond the body outline) were dissected from four specimens. Samples were mounted on aluminum stubs with carbon tape. Four samples of soft tissue were embedded in a low viscosity resin (Ted Pella Inc.) in a Memmert vacuum oven at 60–70 °C until the desired hardness was achieved (48–96 h). Two sediment samples were dehydrated in the same vacuum oven at 60 °C for 24 h, embedded in the above resin at 60 °C for 72 h, polished using a graded series of polishing plates (finishing with 1 µm diamond paste on silk) and mounted on Al stubs with carbon tape. Selected soft tissue and sediment samples were sputter coated with Au. Samples were analyzed using a JEOL IT100 VP-SEM at an accelerating voltage of 5–20 kV and a working distance of 6–10 mm for SEM images and at 10 kV and a working distance of 10 mm for EDS maps and spectra. Acquisition times of 30–45 min and a dwell time of 0.2 ms were used for EDS maps and an acquisition time of 120 s was used for EDS spectra.

### X-ray diffraction (XRD) analysis

16 samples of sediment were selected for analysis. These samples are all dark in color and were distinguished from the soft tissues as they were collected from regions usually beyond the body outline. The samples are from 11 resin slabs, representing four stratigraphic intervals (nine anuran sites). For each sample, a small amount (0.1–2 g) of sediment was powdered manually with a sterile agate pestle and mortar. XRD analysis was performed using a PANalytical Empyrean Series 2 microprobe with a Co X-ray tube, a PIXcel1D detector and a single crystal Si substrate.

### Electron microprobe analysis (EPMA)

One sample of soft tissues from the torso of specimen GMH CeIII-4936a-1932 was embedded in EpoThin 2 epoxy resin (Agar Scientific Ltd.), polished, carbon coated and analyzed with a CAMECA SX-5 FE electron microprobe. Certain measurements used a 5 nA beam with 10 µm spot size and a time-correction applied to Fluorine because of unstable count rates. All other measurements used a 2 nA beam and 10 µm spot size in order to minimize beam damage and a time-zero correction applied to all elements.

### Micro-Fourier transform infrared spectroscopy (FTIR)

The sample for EPMA was used subsequently for FTIR analysis. The resin-embedded sample was polished using diamond paste (1 µm grit) to remove the carbon coating and EPMA beam damage. FTIR spectra were acquired from the freshly polished surface with a Perkin Elmer Spotlight 400i FTIR microscope system. An area of 100 × 100 µm was mapped using a spectral resolution of 4 cm^−1^, a spatial resolution of 1.56 µm^2^ and 128 repeats. Spectra were processed post-acquisition using Perkin Elmer SpectrumIMAGE (v. R.1.11.2.0016) and Spectrum IR (v. 10.7.2) software as follows. An atmospheric correction was applied to the dataset and twenty spectra were extracted from a region of the skin and from a region of the embedding resin. A single averaged spectrum was generated for each sample type (skin and embedding resin, respectively); these average spectra were then baseline-corrected using a standard (non-interactive) algorithm and smoothed by a factor of 10.

### Micro-Raman spectroscopy

Samples of soft tissues and sediment were analyzed using a Renishaw InVia Qontor confocal Raman microscope. Analyses used a 50 mW DPSS (diode-pumped, solid-state) 532 nm laser at 1%, 10% or 50% laser strength with a 50x or 100x objective, an exposure time of 1 s or 10 s and 10–100 accumulations per spectrum. For some samples the analytical area was excited with a 30–60 s bleach prior to the acquisition of spectra in order to reduce the fluorescent background.

### Statistical analysis

Standard statistical summary data were calculated using Microsoft Excel. The number of specimens with preserved soft tissues was expressed as a percentage of the total number of specimens (n = 168). Significance tests were performed with PAST (PAleontological STatistics) 4.15^87^ using the independent samples t-test (α level = 0.05). Data were tested for normality and homogeneity prior to conducting the t-test.

### Supplementary Information

Supplementary Information.

Supplementary Table 1.

Supplementary Table 2.

Supplementary Table 3.

Supplementary Table 4.

Supplementary Table 5.

## Data Availability

The datasets used during this study are part of the Supplementary Files. Additional data can be requested from the corresponding author.
