# Supplementary material for: Fossilized anuran soft tissues reveal a new taphonomic model for the Eocene Geiseltal Konservat-Lagerstätte, Germany
Source: Sci Rep. 2024 Apr 23;14:7876. doi: 10.1038/s41598-024-55822-y (PMC11039752; doi:10.1038/s41598-024-55822-y)
Supplement: Supplementary file 1 — Supplementary Information. [file 41598_2024_55822_MOESM1_ESM.docx]

**Supplementary Information for**

**Fossilized anuran soft tissues reveal a new taphonomic model for the Eocene Geiseltal Konservat-Lagerstätte, Germany**

Daniel Falk*^1,2^, Oliver Wings^3^, Richard Unitt^1,2,4^, Jon Wade^5^, Maria E. McNamara^1,2^

^1^School of Biological, Earth and Environmental Sciences, University College Cork, Distillery Fields, North Mall, Cork T23 TK30, Ireland.

^2^Environmental Research Institute, University College Cork, Lee Rd, Cork T23 XE10, Ireland.

^3^Natural History Museum Bamberg, Staatliche Naturwissenschaftliche Sammlungen Bayerns, Fleischstraße 2, 96047, Bamberg, Germany.

^4^Copper Coast UNESCO Global Geopark, Knockmahon, Bunmahon X42 T923, Ireland.

^5^Department of Earth Sciences, University of Oxford, South Parks Road, Oxford, OX1 3AN, United Kingdom.

**Contents:**

Supplementary Note: 1. Sediment analysis

2. Influence of calcium- and carbonate-rich waters on soft tissue preservation

Supplementary Figures 1–5

Supplementary Tables 1–5

References

*Supplementary Note:*

1. *Sediment Analysis*

Most of the Geiseltal anurans are conserved on resin slabs, where original sedimentary matrix is usually preserved as patches of a dark brown to black homogenous material; these patches are usually a few square millimeters wide and 1–2 mm thick (Fig. 1d). Sediment patches are usually more abundant, larger (a few square centimeters wide) and thicker (a few millimeters thick) where specimens are conserved on gypsum and in paraffin wax. The sediment is brittle and shows polygonal cracking, where fragments can show a vitreous, coal-like fracture surface.

Specimen GMH XXXV-11-1970 is conserved in a slab of host sediment (approximately 10 x 10 cm) and stored in water. The sediment is homogeneous, dark brown to black, fine grained, and shows mm-scale lamination (Supplementary Fig. 5). Laminae are planar and continuous. The splitting surface reveals several macroscopic plant remains (i.e., leaves) adjacent to the specimen, but there is no preferential orientation of the plant material and no sedimentary clasts or grains are visible; there is, therefore, no evidence for substantial sediment input or water flow.

Raman spectroscopy of powdered sediment samples of GMH XXXV-11-1970 and GMH CeI-4947-1930 failed to identify minerals; the sediment samples showed evidence of thermal damage even at extremely low laser power, which is consistent with a sediment composition dominated by organic matter. EDS spectra of five powdered samples show major peaks for oxygen and sulfur; some spectra also show a minor peak for calcium. XRD analysis of 16 samples failed to yield quantitative mineralogical data, most likely due to the organic-rich nature of the sediment, but detected quartz, aragonite, calcite, hydroxyapatite, gypsum and illite/smectite in various samples at concentrations close to detection limits (Supplementary Table 5). The minor quantities of quartz present may reflect minor sediment input; calcite and aragonite may derive from precipitation and hydroxyapatite may derive from phosphate minerals in the sediment or bone fragments. Gypsum and illite/smectite probably represent contamination during conservation. Although the sediment samples are from anuran specimens that represent five fossil excavation sites and four stratigraphic intervals, there are no major differences in composition among the samples (as evidenced by SEM, EDS, XRD and microscopic analysis).

SEM analysis of sediment samples from GMH XXXV-11-1970 reveals micron-scale lamination. Individual laminae are highly anastomosing and laterally discontinuous, with alternating microlaminae that vary in tone in backscatter electron images. These variations in tone correspond to variations in oxygen and calcium in EDS maps (Supplementary Fig. 5), suggesting variation in the content of calcium carbonate. The sediment hosts irregularly dispersed, irregular to oval structures 10–150 µm long, that deform the surrounding laminae. The structures show a porous texture and are enriched in sulfur.

The fossil-bearing sediments at Geiseltal have been characterized as “Weichbraunkohle” [soft brown coal] (Pickel and Wolf^[1]^, p. 482) rich in huminite (52–82%) with a mineral-bituminous groundmass (6–31%) and minor mineral content (<4%). This previous study reported a mineral-bituminous groundmass with a high organic content, a calcium carbonate bearing mineral-bituminous groundmass and low mineral content^[1]^, as in our analysis of GMH XXXV-11-1970. Clearly, during deposition, input of organic debris was higher than for siliciclastic sediment, suggesting deposition in a low-energy regime characterized by low pH and sedimentary anoxia^[1]^, with episodic variation in environmental conditions, thus forming fine macro-lamination. The highly anastomosing, discontinuous, micron-scale lamination (Supplementary Fig. 5) resembles liptinite macerals in coals, that derive from algae (lamalganite)^[2,3]^. The oval porous structures likely represent coal macerals, i.e., sclerotinites^[1,4]^ and/or liptinites^[1,2]^.

1. *Influence of calcium- and carbonate-rich waters on soft tissue preservation*

Preservation of the Geiseltal anurans has been previously linked to an influx of calcium- and carbonate-rich waters derived from Triassic sediments at the southern border of the Geiseltal basin^[5]^. These fluvial waters are considered to have raised the pH of the Geiseltal lakes, promoting preservation of both the skeletons and soft tissues of the fossil vertebrates^[5–7]^. There is, however, no evidence for calcium carbonate precipitates in association with the anuran soft tissues. The anuran E-K layer is replicated in calcium phosphate. Our study shows that the phosphate source and precipitation were localized to the anuran skin and were therefore independent of environmental influences.

*Supplementary Figures*


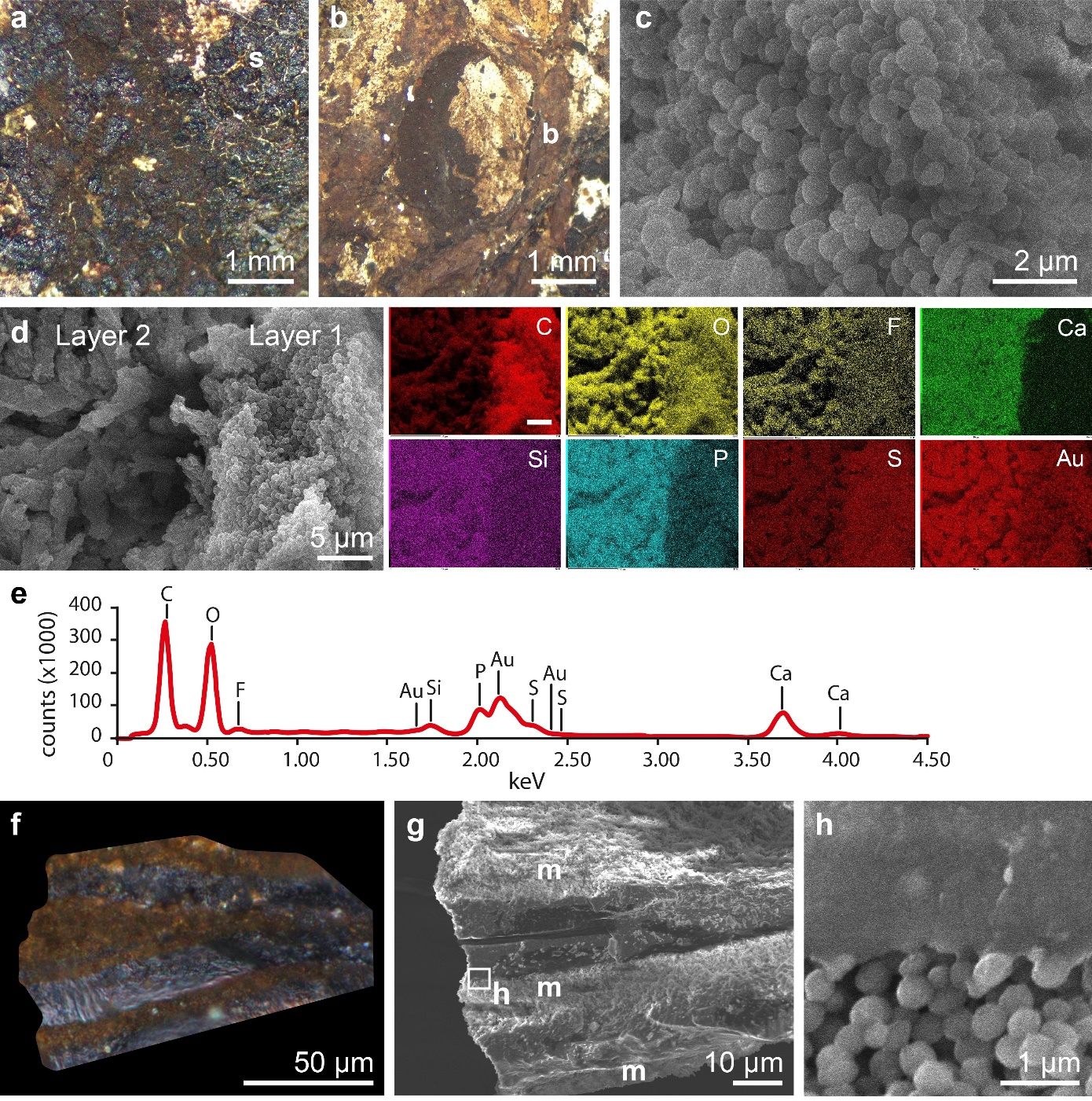


**Supplementary Figure 1:** Layer 1, interpreted as a carbonaceous layer that contains melanosomes. (**a**–**b**) Light micrographs of the dark brown material of Layer 1. (**a**) Region shown reveals (dark brown) Layer 1 next to (black) sediment and is from the inner thigh of GMH CeIII-6748-1932 (Anura indet.). (**b**) Eyespot of GMH CeIII-6733-1932 (Anura indet.). (**c**) Scanning electron micrograph showing melanosomes of Layer 1 in the torso of GMH CeIII-6698-1932 (Pelobatidae). (**d**) Secondary electron micrograph and EDS maps of the junction between Layers 1 and 2 in GMH CeIII-6698-1932, where the melanosome-rich Layer 1 overlaps fibers of Sublayer 2b. Layer 1 is enriched in C and, to a lesser extent, S; Layer 2 is enriched in P, Ca and, to a lesser extent, F. Si possibly represents sediment underneath Layer 2; scale bar is 5 µm. (**e**) EDS spectrum of region shown in (**d**); see Methods for analytical parameters. (**f**) Light micrograph of a vertical cross-section of Layer 1, showing melanosome-rich bands (brown) and melanosome-poor bands (black). Sample is from the torso of GMH CeIII-6698-1932. (**g**–**h**) Secondary electron micrographs of sample in (**f**). (**g**) Bands rich in melanosomes and bands with few melanosomes. (**h**) Detail of inset in g, showing junction between zones where melanosomes are abundant (lower half of image) and rare (upper half of image). b, bone, m, melanosomes, s, sediment.


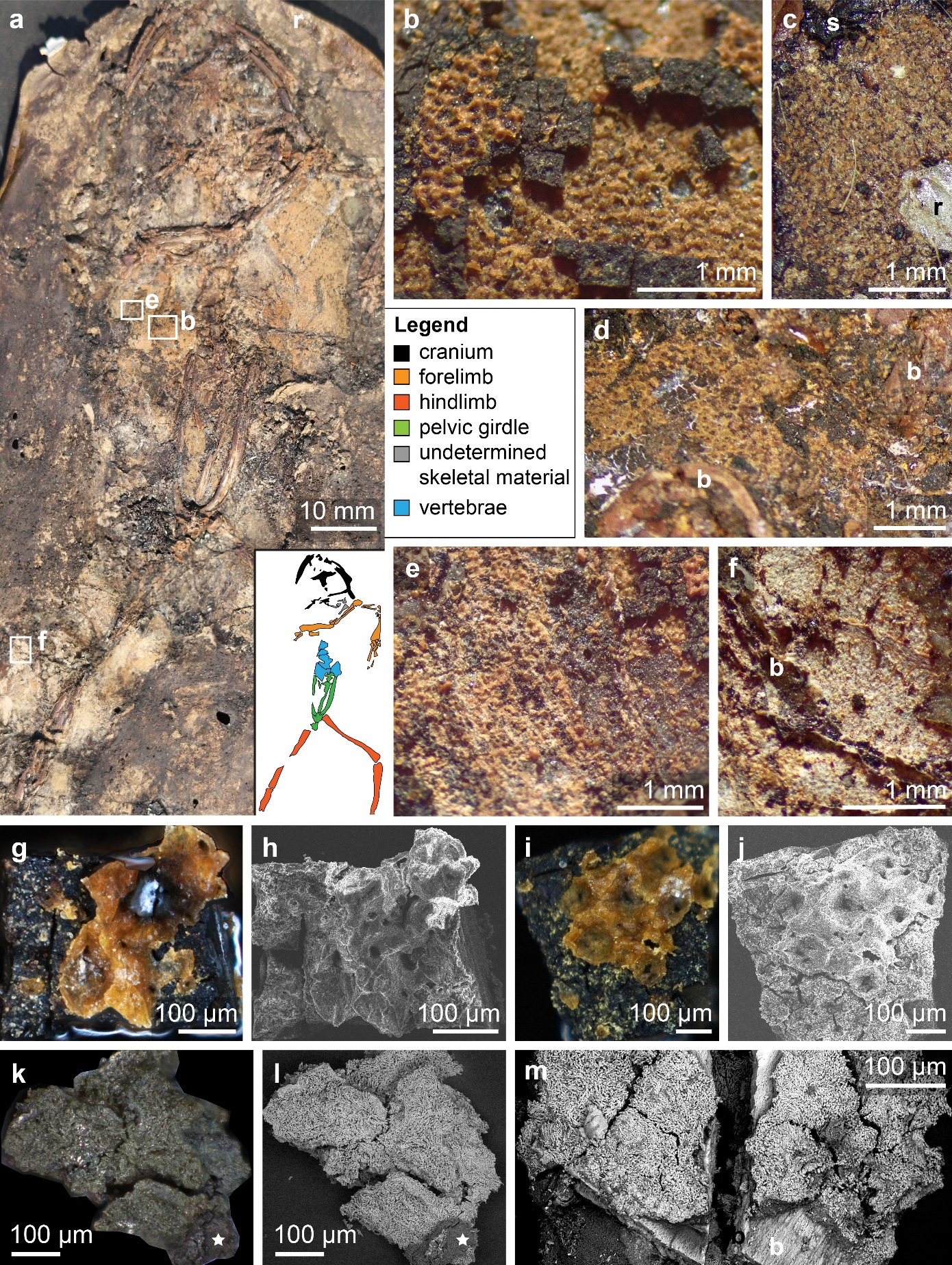


**Supplementary Figure 2.** Layer 2, interpreted as the mid-dermal Eberth-Katschenko (E-K) layer. (**a**) Photograph of specimen GMH CeIII-4936a-1932 (Pelobatidae) showing extensive pale yellow- to orange-colored patches of Layer 2 in the cranium, torso, abdomen and limbs. Insets show color-coded line drawings of the bones. (**b**) Detail of region in (**a**) showing perforations in Layer 2. Layer 2 occurs in two layers, separated by a thick, blocky, dark brown material (Layer 1). (**c**) Perforations in Layer 2, in the abdomen of GMH CeIII-4990-1932 (Pelobatidae). (**d**) Perforations in Layer 2, in the torso of GMH CeIII-6738-1932 (Anura indet.). (**e**) Detail of region in a, showing different taphonomic states of Layer 2: orange with perforations (well-preserved E-K layer) and pale and structureless (poorly preserved E-K layer). (**f**) Detail of a, showing pale structureless Layer 2 (poorly preserved E-K layer). (**g**) Light micrograph shows patches of orange Sublayer 2a on black Layer 1 of specimen GMH CeIII-4936a-1932. (**h**) Secondary electron micrograph of (**g**). (**i**) Light micrograph showing patches of orange Sublayer 2a with honeycomb texture, overlying black Layer 1 of specimen GMH CeIII-4936a-1932. (**j**) Secondary electron micrograph of (**i**). (**k**) Light micrograph of GMH CeIII-6698-1932 (Pelobatidae) showing a small patch of Layer 1 (star) and extensive Layer 2 (Sublayer 2b) in ventral aspect. (**l**), Backscatter electron micrograph of (**k**) shows Layer 1 (star) on Sublayer 2b. (**m**) Backscatter electron micrograph of succession of preserved soft tissues and bone in GMH CeIII-6698-1932 from top to bottom of the sample: Layer 2 (Sublayer 2b), Layer 1, bone, Layer 1, Layer 2 (Sublayer 2b). b, bone, r, resin, s, sediment.


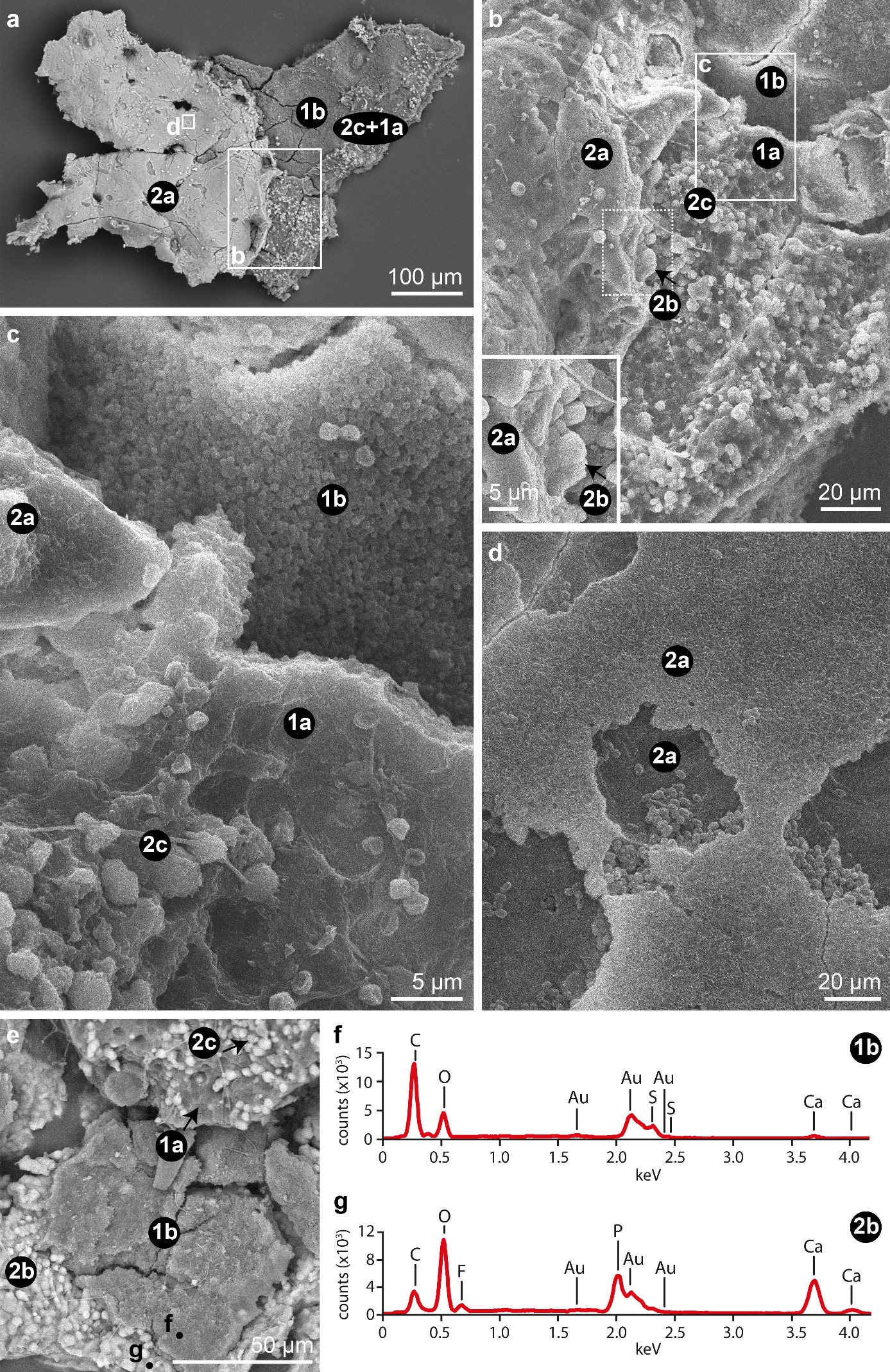


**Supplementary Figure 3.** Succession of soft tissue layers in the Geiseltal anurans. (**a**–**d**), Electron micrographs of GMH CeIII-4967-1932-B6 (Pelobatidae) show soft tissue Sublayer 1b (melanosome-rich), Sublayer 1a (melanosome-poor), Sublayer 2c (globules, that form molds in Sublayer 1a), Sublayer 2b (fibrous) and Sublayer 2a (amorphous). Not all soft tissue layers are continuous across the entire area of the sample. (**a**) Backscatter electron image showing soft tissue layers. (**b**) Secondary electron image of region indicated in (**a**), showing successive preserved skin layers. Close-up in bottom left corner shows fibers of Sublayer 2b overlain by amorphous Sublayer 2a. (**c**) Secondary electron image of region indicated in (**b**). Sublayer 1b is separated by Sublayer 1a from Sublayer 2c. (**d**) Secondary electron image of region indicated in (**a**). Dimpled outer, amorphous surface of Sublayer 2a with local clusters of ovoid microbodies. (**e**–**g**) Backscatter electron image of a region in CeIII-4967-1932-B6 and EDS point spectra of regions (**f**) and (**g**) in (**e**); Au peaks are from the gold sputter coat; see Methods for analytical parameters. (**e**) Successive layers showing irregular globules of Sublayer 2c underlain by homogenous Sublayer 1a, melanosome-rich Sublayer 1b, and in turn fibrous Sublayer 2b. (**f**) EDS spectrum of Sublayer 1b Ca peak reflects X-ray signal from the underlying Sublayer 2b. (**g**) EDS spectrum of Sublayer 2c.


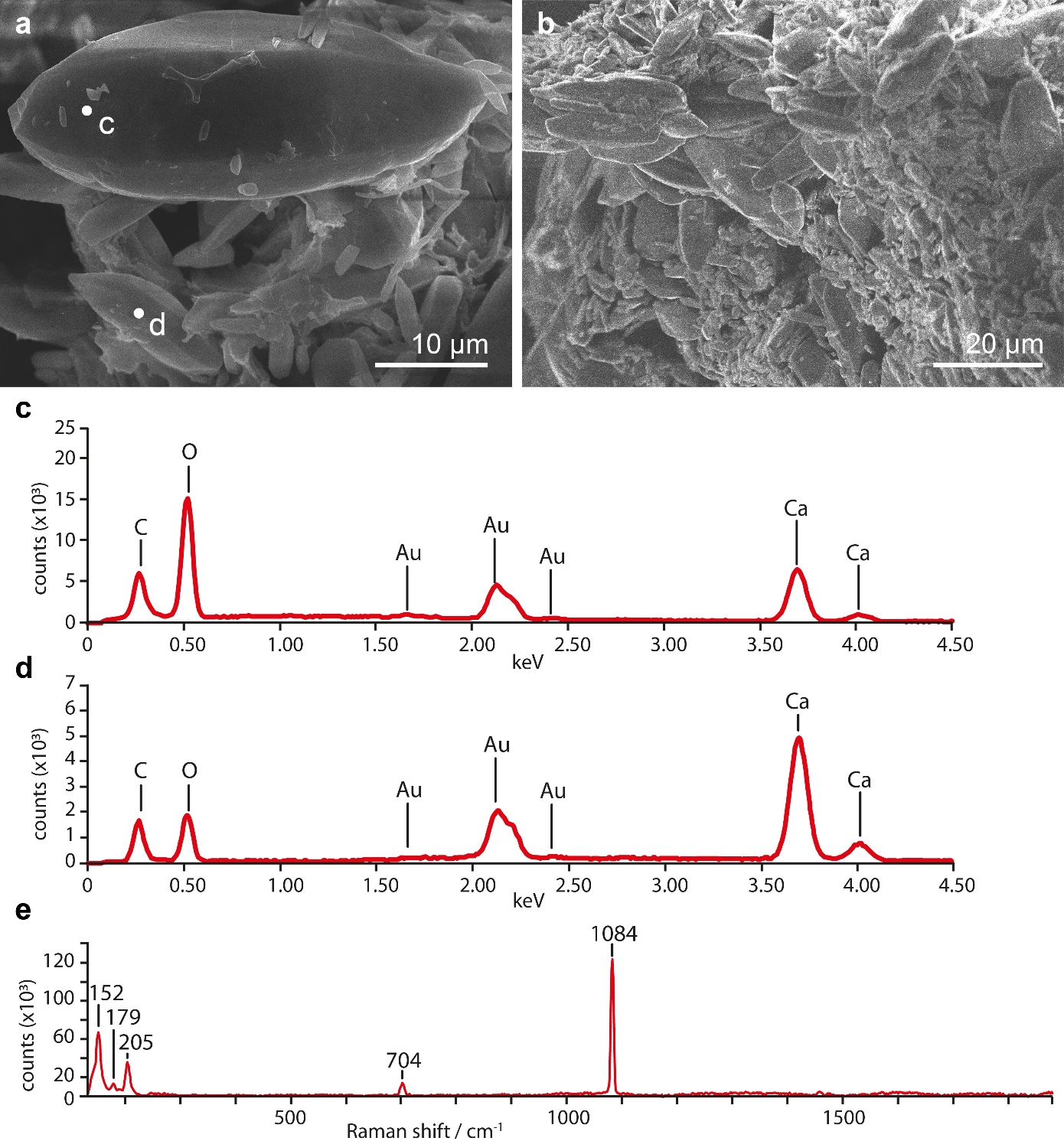


**Supplementary Figure 4.** Aragonite crystals in the cranium. (**a**), Secondary electron image of euhedral, micron-scale crystals in the white material in GMH CeIII-4967-1932 (Pelobatidae). (**b**) Secondary electron image of white material in the cranium of GMH CeIII-6698-1932 (Pelobatidae). Points in (**a**) indicate the locations where the EDS point spectra in (**c**) and (**d**) were collected. (**c**–**d**), EDS point spectra from points indicated in (**a**). Sample is Au coated. (**e**) Raman spectrum for sample shown in (**b**) with a major peak at 1084 cm^-1^ (v_1_CO_3_^2-^) and minor peaks at 152 cm^-1^, 179 cm^-1^, 205 cm^-1^ and 704 cm^-1^ (v_4_CO_3_^2-^). This spectrum is characteristic of aragonite^[8]^.


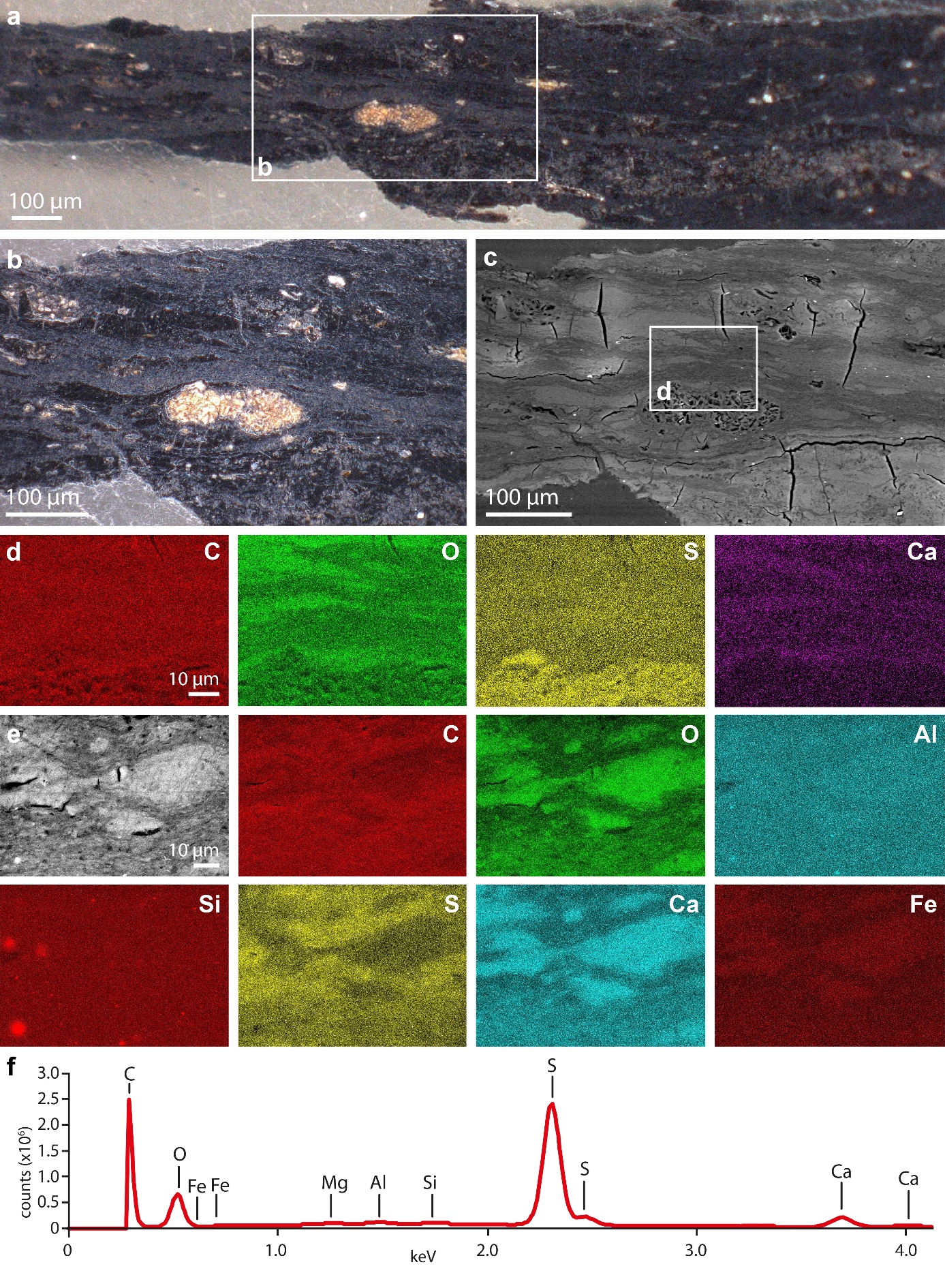


**Supplementary Figure 5.** Analysis of the host sediment associated with GMH XXXV-11-1970 (Anura indet.); see Methods for analytical parameters. (**a**–**b**) Light micrographs of a vertical polished section through the sediment. (**c**) Backscatter electron image of (**b**) showing alternating dark and bright, laterally discontinuous laminae (probably liptinite coal macerals) surrounding porous structures interpreted as isolated sclerotinite coal macerals. (**d**) EDS maps of the region shown in (**c**). (**e**) Secondary electron micrograph and EDS maps. Bright areas in the micrograph are enriched in O, Ca and to a lesser extent, Fe; dark areas are enriched in S and to a lesser extent, C; localized hotspots of Si likely reflect contamination during polishing. (**f**) EDS spectrum from area shown in (**e**).

*Supplementary Tables*

**Supplementary Table 1**. Master dataset with soft tissue data for the Geiseltal anurans. Data are shown for each of 168 specimens used in the study. Hyphen (-) indicates an unknown date of specimen collection (column D) and/or lacking stratigraphic data (column L). Abbreviations in columns G and H indicate the anatomical region where Layers 1 and 2 occur. “mold” refers to an external mold of E-K layer tissue texture in resin or wax. “skeleton” refers to a specimen comprising articulated bones from most or all of the cranium, torso, abdomen and/or limbs. “disarticulated bones” refer to skeletal elements that are not in their original relative body positions *in vivo*. “x” denotes where Layer 1 and/or Layer 2 is/are present but lack anatomical data (e.g., due to the unconfident determination of skeletal elements). “#” denotes where a feature is present. “?” indicates uncertainty regarding the presence/absence of a feature.

Abbreviations. Ce, ‘Cecilie’ pit; IL, ‘Geiselröhlitz’ site; GMH, Geiseltalmuseum Halle; indet., indeterminate; Leo, ‘Leonhard’ pit; LMC, Lower Middle Coal; LUC, Lower Upper Coal; MC, Middle Coal; MUC, Middle Upper Coal; Nw, ‘Neumark-West’ pit; N/A, not applicable; UHM, Upper ‘Hauptmittel’ (‘Hauptmittel’: intercalated sandy and silty successions); UMC, Upper Middle Coal; XXXV, Neumark-Süd pit (NsXXXV). Anatomical region: cr, cranium; eye, eyespot; ext, beyond the body margin; fl, forelimbs; fp, forelimb phalanges; hl, hindlimbs; hp, hindlimb phalanges; pel, pelvic girdle; tor, torso; vc, vertebral column.

**Supplementary Table 2.** Historical description, interpretation, and mode of preservation of the soft tissue features of the Geiseltal anurans.

**Supplementary Table 3.** Raw data for EPMA of sample GMH CeIII-4936a-1932-A1 (in resin).

**Supplementary Table 4.** FTIR peak assignments for the calcium phosphate mineral phase analyzed in a sample of the E-K layer from specimen GMH CeIII-4936a-1932.

**Supplementary Table 5.** Raw data for X-ray diffraction bulk mineralogy analysis of sediment associated with Geiseltal anurans. Sample 1305-S1 represents a conservation material comprising mainly illite/smectite and muscovite with a minor composition of quartz, chlorite, feldspar and gypsum.

*References*

1. Pickel, W. & Wolf, M. Kohlenpetrographische und geochemische Charakterisierung von Braunkohlen aus dem Geiseltal (DDR). *Erdöl, Kohle, Erdgas, Petrochem.* **42**, 481–484 (1989).

2. Pickel, W. *et al.* Classification of liptinite – ICCP System 1994. *Int. J. Coal Geol.* **169**, 40–61, http://dx.doi.org/10.1016/j.coal.2016.11.004 (2017).

3. Gallwitz, H. Kalk, Kieselsäure und Schwefeleisen in der Braunkohle des Geiseltales und ihre Bedeutung für die Fossilisation. *PalZ* **29**, 33–37 (1955).

4. Taylor, G. H. & Cook, A. C. Sclerotinite in Coal - its Petrology and Classification. *Geol. Mag.* **99**, 41–52, https://doi.org/10.1017/S0016756800057113 (1962).

5. Krumbiegel, G., Rüffle, L. & Haubold, H. *Das eozäne Geiseltal: ein mitteleuropäisches Braunkohlenvorkommen und seine Pflanzen- und Tierwelt*. 227 pp. (A. Ziemsen Verlag, 1983).

6. Haubold, H. & Thomae, M. Stratigraphische Revision der Wirbeltierfundstellen des Geiseltaleozäns. *Hallesches Jb. Geowiss.* **15**, 3–20 (1990).

7. Voigt, E. Weichteile an Fischen, Amphibien und Reptilien aus der eozänen Braunkohle des Geiseltales. *Nova Acta Leopold. NF* **5**, 116–142 (1937).

8. Urmos, J., Sharma, S. K. & Mackenzie, F. T. Characterization of some biogenic carbonates with Raman spectroscopy. *Am. Mineral.* **76**, 641–646, <http://www.minsocam.org/ammin/AM76/AM76_641.pdf> (1991).
